# Supplementary material for: Food insecurity in South Indian households with TB during COVID-19 lockdowns and the impact of nutritional interventions: A qualitative study
Source: PLOS Glob Public Health. 2025 Apr 9;5(4):e0004242. doi: 10.1371/journal.pgph.0004242 (PMC11981200; doi:10.1371/journal.pgph.0004242)
Supplement: S1 Data — Material not related to the focus of this manuscript has been removed. (DOCX) [file pgph.0004242.s001.docx]

# **Focus Group Discussion – females only**

- **Medical social worker:** [59.29] Yes, okay. Almost last question, okay? In this Corona time, isn't it 2019? Two thousand and nineteen Corona has come? At that time, everything was on lockdown. So, due to the lockdown, everyone has suffered and felt discomfortable, without going to work.
- **Medical social worker:**  Everyone has all the work in the village. okay? So, how did you cope at home during the lockdown? How was the income? How come you managed?

**Number one,** tell me. Do you understand what I am I saying?

- **Medical social worker:**  In Corona time, we can’t go to work from home. We don’t have income. Then, without income, how did you manage, what kind of problems were there in your family?

**Number 01:** It was very difficult. We have suffered very much.

- **Medical social worker:** [1.00.12] How to cope?

**Number 06:**  however, we are suffered a lot since we have to eat and we have to manage and become his own.

**Number 02: [1.00.22]** Because no one is going to eat properly since because of no job at that time. Now let's pawn all the little jewels in the house and make adjustments and we have it, sir.

- **Medical social worker:** [1.0030.] So, have you recovered all the jewels that you had?

**Number 04:** Yes sir. Now that he is going to his house, let's restore all the jewels, sir.

- **Number 03:** Tell me. How did you manage then?

Sir, we used to manage the things that were given in Ration and also you have given.

- **[1.00.56] number 04:** No one at home was going, only I was going to his work, sir, so we used only Ration items, at that time, we managed that way, and then all the jewelry was pawned.
- **Number 06: [1.01.20]** We also pawned all the items at home and ate them. Then we managed to eat the missing items from Ration shop.
- **Medical social worker:** [1.01.20] Number 05: how did you manage?

**Number 05:** Sir, I was going to work as a mason then.

- **Medical social worker:**  So, you got all the brick work isn’t it?

**Number 05:** At that time, I was going to a mason for a couple of months and then I used to have some porridge, sir.

- **Medical social worker:**  Number 07: [1.01.41] Can you tell me?

**Number 07:** I have been struggling a lot since then, sir

- **Medical social worker:** [1.01.50] Even then, how are you now, sir?

**Number 07:** No worry now sir.

- **Medical social worker:** [1.01.56] At that time, most of us used to borrow money from the shops, but we are stuck. Did you get all the groceries you needed in the shops at that time?

[1.02.14] How did you manage, tell me that all the shops were not available at that time?

**Number 06:** At that time, all the products were very expensive. Sir, it was very difficult for me to do with such a price.

- **Number 05:** [1.02.26] Then we have a situation to buy things so that we have spent on it.
- **Medical social worker: [1.02.38]** For example, tell me how much of the milk packet could be sold at the right time and how much has in the other time?

**Number 05:** Then everything was too much. I don't know how to say it.

- **Medical social worker:** [1.02.43] What do other people think?

**Number 04:** All the vegetables are very expensive, sir.

That's why I didn't buy any vegetables, sir. I can't buy anything like before, except for the onion, tomato, chilly, brinjal, etc. Even if you want to buy anything now, you will buy it in 3-4 days.

- **Medical social worker:** [1.03.08] Did you get the essentials you need during the lockdown?

May it be milk or rice or vegetables or any other material?

**Number 04:** Not available sir.

- **Number 03:** You have saved all the items since we need to have them for tomorrow. We have used them. Mostly porridge only we used more sir.

Let's put the porridge for today, sir, tomorrow.

- **Number 04: [1.03.29]** Sir, while we are preparing the gravy we use to make it in excess so that we can keep it using for the next days.

That's how we managed it sir.

- **Medical social worker:**  Number 3 Is it not what she said?

Do you understand what I am asking? Now, for example, let's say that you normally cook a kilo of rice, but in those times how much you have cooked like half a kilo of rice like it.

**Number 04:** As Ration rice was available, so we never reduce the amount of rice usage for cooking. But all the gravy fries are very chewy, sir. Even if there is any left over, let's eat it with porridge.

**Number 05:** My son was not here at that time and went to plumbing bore work. We use to cook a quarter of a kilo of splitted rice and we eat it like it, sir.

- **Medical social worker:** [1.04.30] Okay.

We have observed so many things from you. All these things are very useful. Let's share all this with our officers in JIPMER and think about how we can develop everything in future.

Thank you very much for taking your time for us.

Everyone is having a good conversation.

# **Focus Group Discussion – males only**

- **Medical social worker-** Sir, this is last question, okay? I want to talk a little bit about everything. Let's talk about it in detail.

Okay, come on this corona, period has not come, corona in 2019, has been a huge problem. For that, we have come and solved all kinds of problems. In these Corona times, shouldn't we put all the lockdowns during that corona period?

- Medical social worker-Is it possible to go and buy things at that time?

What if there is a situation where you can't come and go?

**Number 01**-We can't eat properly and so we have taken a loan, the amount gets over.

But at that time even if we have the money we can’t even cook and eat properly since because of unavailability of the required things .We can’t eat properly .It was just like eating food, everything like that, there was no store at all.

**Number 03 -** Department store is not there. But, the small shop next door had everything. We use to buy things there only.

- **Medical social worker:** What about number 06?

**Number 06:** We use to bought the vegetable from the same place. The shop never opened outside. So, we bought and made it from the locality place where the product is available.

- **Medical social worker-**For example, the shop is very busy right now, isn't it? Let's take a packet of milk price as an example. Is the price of a packet of milk higher than the usual price during those periods and during the lockdown period? It is something, if you remember it, you can tell about it.

**Number 01-** Milk packet, now normally, even if you buy it for ten rupees, but no packet will be easily available at that time. Not all these ten rupees, they use to sell as fifteen rupees milk packet. Half a liter packet, now all rates have gone up by ten rupees. At that time, it's very complicated, they come only for one day. There is a lot of buying and selling, and the rate is high, and the price of everything is high, sir.

**Number 02- At those time everything was very costlier only sir.**

**Number 02-**The cost of a milk packet is more than that. Here too.

- **Medical social worker-**Number 03- Will you ask me back? In that lockdown period, the prices of everyday items that can be bought are too high? So, what is the most important thing?

The price of milk was high. According to other things, the price of onion was high. And other things also will be very high.

**Number 07-** You? Number 07. The price of milk is also very high

**Number 05,** you tell me. Everything was costlier at that time only sir.

Pondicherry Government will not give anything at those times. They have did nothing for the people. But the Delhi government gave it. The Prime Minister gave rice to fifty thousand to 20000 families. That's gone, Corona is different. No one gives anything from Pondicherry Govt.

Okay. Work is for us today. It is our responsible to go to work and we have to take the responsibility of that. They have given the Ration rice

We have bought that rice from there like Fifty kilos of rice. At that time, otherwise, we would be hungry.

Now if you think of the Prime Minister, he is proud like what did he give?

Okay, okay.

**Number 07-** You tell me. Like I said, milk is very expensive. There is no other way for them. Because it was very scarce. So, this is a situation and necessity where we have to buy it.

- **Medical social worker:** Okay, okay. Thank you very much everyone.

I asked you about everything. Why, if I asked a few things repeatedly, it was because I wanted to get everyone's opinion. For example, only things that number 02 said come up, and he may have said them personally.

As far as I am concerned, if we are talking about six or seven people, are their opinions and your opinion, correct? Talk about the contradiction, in about a quarter of an hour, it's been an hour, okay?

So, everything you talked about came through and we shared a good opinion and what you said, everything came and would have been useful for us, surely. We come to the future and you said, there are little things We are making efforts to improve all that. Didn't we talk about something else? Beyond that, is there anything else you would like to say?

For example, say number 01 now. Nothing else, sir. The same is correct. Number one. This is the correct answer if you understand it. It's okay, sir.

**Number 04** is let's stay together or not? What do you think about that? Number 04: Almost, our four. If you give for six months, well, even if you give it for extra two months, we will be a little fine and have a little chance to run.

- **Medical social worker-**Well, the end has come, what are our number 04 saying now is, six months we are giving the food items. That food item can be added for two months and then eaten for eight months.
- **Medical social worker-**What is the opinion about it? Let's conclude. No, if it is enough for six months, two months for eight months, then we will pay for that. I think that eight months will be good.
- **Medical social worker -** Okay, now when we are discussing, it now, we are talking about whether we give for six months or not. So, if you come and see the important thing, if you give us nutritional products instead of the food we give you, you will be fine.

Beyond that, if we give cashews, almonds, etc., it will be useful for them. So, you have come to this group and shared so many things. So, you are very welcome to everyone. Okay. Thank you so much everyone. Thanks

# **Individual Interview: HHC-F-1**

I: hmmm okay. During the corona pandemic in 2020, they imposed lockdown for 4 months. During that period how difficult was it financially for you. How did it affect you. Was there some sort of income.

P: no sir. There was no income.

I: there was no income?

P: all of us were at home only. Husband , me and my son were at home only. We did not go anywhere and were in home only for few days. It was very difficult for food. We suffered a lot.

I: did you get any loan to handle that?

P: we did not get any loan at that time.

I: you did not get any loan? Then how did you handle it?

P: we cooked whatever was available at home and managed.

I: So you had something at home. Did you have sufficient at those times or did you borrow from neighbors.?

P: no no not like that.

I: they would have not opened the shops at that time. In case the supplies were over, what would you do?

P: we managed with hat was there at home. If not we would have bought from the local stores.

I: did they not close the local shops.

P: no no they did not close. It is in interiors of the village.

I: okay. Was everything you need available there. Like rice, pulses, biscuits, vegetables etc. or only few items were available .

P: no no everything was available. Mostly everything was available but only fish and meat was not available.

I: okay okay.

P: But all other grocery items were available.

I: ok ok did you have money to buy all this.

P: yes we had. We had saved earlier. It was little difficult. We managed with porridge. It went on like that.

I: so you did not get any loan. You managed with the what you had saved.

P: yes yes it was saved before.

I: so starting from May 2020, how many months did it affect without income?

P: two to three months it was difficult.

I: did you not go to work for three months?

P: we did not go to work. We were at home. We only ate and slept.

I: After the restrictions were reduced , when people were allowed to work half a day, did you receive any income during that time?

P: Hmm yes we received.

I: Did they give entire salary.

P: they gave entire salary. There was no deficits in that.

I: All the information given by you will be very useful for our research. Thanks a lot.

# **Individual Interview: HHC-F-2**

I: Good good. All your suggestions and comments were very good. You discussed with us regarding the delivery of food supplies. Now let me ask a few questions about how you dealt with Covid-19 pandemic. We will be happy if you share your opinions regarding this.

How was your income affected during the pandemic?

P: No one went for work from my house. My father never used to go to work. We have goats at home. We take care of it and sell it for business. However, because of lockdown no one purchased the goats. It was very difficult for us. The moment you came, it was very helpful for us.

I: What would you have done if these food supplies were not given to you?

P: Dad would have gone to work. Even I might have gone for work in a nearby company. We may get weekly wages if we go for work. We would have used that money for running the family.

I: You have stated your situation during the lockdown. What would have happened if things were not provided by us? Would you borrow?

P: Yes. I would have borrowed. My dad definitely would’ve borrowed money. We might have sold all our hens and goats in the house for getting money.

I: Have you sold things at your house?

P: Yes. We ate food by selling things.

I: Would you borrow money for just food supplies or even for medical purposes?

P: Even for medical expenses we would’ve borrowed.

I: Was it needed for medical expenses?

P: Initially we went to private hospitals when my mom was sick. Later on we consulted at government hospitals.

I: You mentioned that you did not have any income during the lockdown period. What was your income before and after the pandemic?

P: We work daily in the cashew farms and get Rs. 200 as daily wage. But after this lockdown, shops and companies were closed. So at that time, we ate only leftover rice.

I: Was it because of no income that you had leftover rice?

P: Yes, it was because of no income.

I: How much did you earn in a day?

P: I had earned Rs. 200 in a day.

I: Did losing your income reduce the number of meals you have in a day?

P: Yes it did.

I: To what extent did it reduce?

P: I ate only twice a day.

I: During lockdown, were you able to go out and get things?

P: No I couldn’t.

I: Why?

P: Because we were not allowed to go out. The shops were not open. And we did not have money. We adjusted with whatever we had.

I: We are very happy that you shared your valuable opinions. It will help us for future research purposes. Thank you so much.

P: thank you.

# **Individual Interview: HHC-F-3**

I: Till now we have been speaking about food supplies. Now I want to know about how corona affected your income? So from March we had been affected from corona. So did your income get affected?

P: Before corona I used to earn around 140rs or 120rs. I used to go to work at good night company, I also worked at a company in villianur.  Now because of corona I cannot go anywhere. It is very difficult. I have to send my son to college , the travel and food take almost Rs 150 for a day.I used to buy betel leaves for 15rs, I could not go and buy that. I have to send my son to college. My husband also needs money for medicine, hospital visit he keeps having body pain. We suffered without money because of that. I used to go to work for 30 days.

I: so you used to go to work when there was work. Were you able to go to work during this period?

P: No I was not able to. The corona virus was spreading. I had breathlessness. We faced loss because of it

I: How much did your income reduce because of it?

P: I would have stayed at home for six months to one year. Around 3000 per month was reduced. Almost a loss of 20000rs I faced.

I: How did you change your expenditure during this period? Were there changes in your daily expenses because of that?

P: My husband used to drink alcohol daily. He stopped having alcohol, he used to smoke cigarettes. He stopped that.

I: so because of no income he stopped drinking alcohol and smoking cigarettes.

P: yes

I: So how did you manage. You would have needed that 3000rs per month right. Did you borrow money or did someone help?

P: My sister supported us. She bought us the soap for bathing. She biught us 10rs soap for my son, husband and me. She bought us paste, powder etc that was useful for somedays. When it got over I managed to brush with salt for a few days.

I: So you managed to reduce the expenses by making few changes?

P: yes

I: So you sister supported you?

P: Yes, I feel bad she does not have husband. She goes to work to earn. Because I am suffering they help me.

I: okay fine. Were you able to go out and purchase the food supplies during lockdown?

P: It was difficult to go out during the lockdown. So the nearby shops we used to buy. They used to sell a 5rs worth stuff for 7 or 8 and blame it on corona.

I: Did you have any savings to utilize during the corona time?

P: I had little rice and toor dal. I had little a wheat flour. We somehow managed to eat without whatever we had.

I: Were you able to procure all the necessary items you need?

P: No. Onion and tomatoes were not available. There would not be the ingredients to cook.

I: okay. Thanks a lot for your opinions and suggestions. This is very helpful to us.

P: thank you.

# **Individual Interview: HHC-F-4**

- **INTERVIEWER:**  [ 39.20 ].Okay. Regarding The **COVID** Condition Since March 2019  To Till 2021  Till Now,There is a Lot Of Unemployment, Lockdown Especially In The First 6 Months Severely ?So Now I Am Going To Ask Some Questions Related To That Now?

For The First 4 Months Of Strictly Followed Lockdown Situation, How Much Did It Affect Your Family In Case Of Work Related Issues Or Financial Issues Or Something Like That?

**HHC :** We Have Suffered A Lot In Terms Of Getting Food For Our Life. We dint Have Job Also .So We Used To  Get Some Food Items From Our Ration Shop And We Adjusted with It. We Couldn’t Even Buy Vegetables Also .We Used To Have Sambar, Moringa Leaves And it was very hard to  Manage In Those Hectic Times.

- **INTERVIEWER:**  [ 40.49]-In Those Times Your Pay got Reduced A Lot, Or You did Not Even Receive Any Salary ?

**HHC :** We dint Have Job That Time. We Have Suffered A Lot And We All Got struck In Our Home For The 6 Months.

- **INTERVIEWER:**   [41.20] .Have You Ever Got A Loan Or Debt From Anyone To Manage In That Hectic situation? If You Don’t Mind Can You  Tell How You Managed During That Period? Have You Reduced The Amount Of Food you consumed In Your Family?

**HHC :** We Have Borrowed  Some Money Around Some 15000  To Buy Some Vegetables And Other Groceries.

- **INTERVIEWER:** [  42.00] In That 6 Months Of Lockdown Situation Most Of The Shops Got Closed And If It’s Opened It Would Be For Some Particular Time ,In That Case How You Managed ?How Have You Faced That situation?

**HHC :** Most Of The Shops were Closed At thatTimes**.** But Evening Only One Hour, It was Opened ,And In That Time We Have To Get Things In A Queue Only.

Many Of Them Rich Ones  Have Money And They Have Purchased Everything In A Huge Amount And We Couldn’t Get The Needful Amount Since We Are Not Having Enough Money To Get Everything And We Have  Suffered A Lot For Food During The **COVID**.

- **INTERVIEWER:**   [42.31]-Was Everything Available In The Grocery Shop In Required Quantity In Those Time Of **COVID** ?

**HHC :** Since We Are In The Queue We Didn’t Get Enough Supplies Since The Rich  Ones In The Previously Bought Most Of The Groceries.

- **INTERVIEWER:**   [42.50]—So You Have   Faced And Overcome A Lot Of Difficulties During The Hectic Period  .As You Already Said ,You Have Borrowed Some Money To Manage The Situation .In That Case How  Much Time you took To Settle Those Debts From Your Side?

**HHC :** As The **COVID** Starts From March 2019 And It has Taken  One Whole Year To Settle Those Debts And Still We Have Remaining 5000 Rupees  To Settle.

- **INTERVIEWER:**  [ 43.13] So Now Also You Are Giving The Money. For Settling These Debts?

**HHC :** Yes Sir ,Now Also We Are Giving Interest  Money  For Every Month. We Have Borrowed Only for Food Expenses Only.

- **INTERVIEWER:  [**44.03]— You Said you have faced difficulties During The **COVID** Time. From That Situation What You Have Learned Or Got Knowledge From Like Savings ,Some things  Like That?

**HHC :** We Never got into This Kind Of Crunch Situation As Like In The **COVID** .I Have Learned To Save Money And Previously Buying Some Groceries In Stock.

- **INTERVIEWER:** [ 44.02]You Have Finely Shared your Experiences And Everything  To Us In A Mannered Way. These Will Help Us To get Better In The Future Times.

Thank You So Much For Your Precious Time With Us and For Sharing Your Ideas And Experiences

**HHC :** Thank You Sir….

# **Individual Interview: HHC-F-5**

I: okay. in the past two years we are struggling with Corona. Because of it there were lockdowns, we were not allowed to step out of home. what kind of difficulties did you face during those times?

P: At that time you gave us the food supplies, it was useful. We did not face much difficulties because of that. No one at home was employed at that point. we cooked and ate from the food supplies you gave us.

I: To what extent was your income affected because of corona?

P: we were all jobless during that time. We borrowed money and ate food. We were borrowing from the nearby store and eating.

I: okay. How much money did you borrow. Were you able to return it?

P: We gave it off little by little.

I: During corona you did not have income, so did that affect your food intake? Did you reduce the quantity of food you consume?

P: no we borrowed and ate. We did not go  without eating food.

I: were you able to get all the food supplies you needed for cooking?

P: Yes they used to open shop now and then, we used to get at that time.

I: we know homes where because of no income they survived on eating porridge. Did you face such situation?

P: No it was not like that.

I: did anyone in your neighborhood or in town help you during that time.

P: no..no one helped.

I: so you borrowed and ate

P: yes we borrowed and ate

I: During the corona time there was a lockdown, were you able to buy food supplies at that time?

P: yes it was available. We used get it from the nearby store.

I: so the stores were open for purchase.

P: It was closed outside. I am talking about the shops in my street. We used to get it from that shop alone.

I: okay. You were able to get everything you need there?

P: yes we got it.

I: okay. What do you think about the quanity of us delivering the food supplies  to  you? is it enough

P: it is enough in fact it is excess.

I: It is more than you need?

P: yes.

I: what do the people at your think about the quantity of the food supplies?

P: They told it correct.

I: did they think it was excess.

P: yes they thought it was excess.

I: Did your neighbourhood question you on who are giving these food supplies or why they are giving?

P: They won't ask us anything. They will speak among themselves.

I: okay. If we gave you these food supplies through your nearby grocery store or through a ration shop will you get it?

P: Yes, we will get it. you can give it General hospital , my husband can get when he visits the place.

I: If we give it through other stores by giving you food coupons?

P: no we don't need to do that. We will get it if it is given there.

I: so if we give to hospital  you can get it from there

P: Yes, we will get it from there.

I: do you think it is better if we give you cash instead of food supplies?

P: it is good if you give it as supplies instead of money.

I: if we put the money in your account will you take it?

P: it will be difficult to go and get the money from the account. It will be good if you have food supplies.

I: if we gave you money would you spend it on other things instead of food supplies?

P: no we will buy only food supplies from that,

I: okay. Thank you for sparing your time for this. You have answered everything well. we are very thankful.

# **Individual Interview: HHC-F-6**

I: Ok mam. You answered very well. During this COVID situation how did you manage. Only two more questions remaining. Lockdown was during march, April 2020, during this lockdown how was your income, how did you manage that.

P: We did not have any income at that time. We sowed paddy. Initially we get money from merchant for sowing paddy and after harvesting they reduce the initial amount which we borrowed from them and they give back the remaining amount which was the only source of income at those time. We were not able to go anywhere else, we get money from merchant.

I: Ok. Before Corona lockdown how was your family income. What all happened…was there any change in income after lockdown?

P: I have two brothers. One of them is taking care of agriculture and the other is working in transport services(Bus). During lockdown there was no bus service, so the only source of income was from the land.

I: So you are borrowing money and taking care of your family?

P: If we are planning to harvest paddy for 50 thousand rupees, we borrow 20 thousand rupees from the merchant. After harvesting, the merchant will take the amount which he lended after clearing all the accounts and he gives back the remaining money to us.

I: Ok. So you will get money for the paddy you are going to grow. Is it like an advance?

P: Yes, its like an advance. Finally we are getting twenty or thirty thousand, after giving back their borrowed amount, which we will be using it for the next sowing.

I: For example, you will be seeing a profit of fifty or sixty thousand a year, so you are getting twenty or thirty thousand as an advance, so how will you be able to manage the situation in future? What doyou think about that.

P: what I felt was…our land is under the control of merchant, so he lends how much ever amount we ask for….and now as the bus services have resumed, my other brother is also going for work, and im also a daily wage construction worker, thats how we manage the family.

I: Did you borrow the amount from the merchant with interest amount?

P: That….like loan….it is like , if we are getting a sum of ten thousand, they will take one thousand as an interest. They will subtract that then they will give the amount. It is like an interest only.

I: So its like a loan right?

P: Yes.

I: During the lockdown time, imagine that you cook around five or six kgs, or it can be two kgs as well. Did you reduce the food that you consumed during that lockdown time?

P: We cany have reduce the quantity and all. We are only harvesting the paddy right. We keep 10 bags of ourselves. And when we turn it to bags we will have 6 or 7 bags. So we do not have problem with the quantity of rice. The problem will be towards the amount which will spent for the gravy. We get the money from merchant in order to manage those expenses.

I: Ok. Were you able to afford meat, fish etc?

P: That all was difficult

I: How did you manage all that?

P: It was difficult to make meat, fish instead we only made sambar, spinach etc, during the COVID time.

I: During the time of lockdown, all the shops were closed. What were the difficulties you faced to buy food products

P: There will be time restrictions that time, it will be open only till 12 in the afternoon so we will go and buy at that time and keep it in our house. We will not be able to manage the expense if we go and buy everyday. If we buy vegetables for about 500 rs for a week, it will be fine. So we used to buy and store it like that. If it is small family, we can manage with even one kg of onion and tomato per week, but as our family is very big, we need to buy at least 4 kgs of tomatoes  and onions each week. My children do not eat without tomato chutney. Whatever hardship we face, we buy it and store it.

I: was the shops open at that time?

P: It will be open only for an hour, at that we should go and purchase it.

I: can you please elaborate on the difficulties you faced for getting food items from the shop within that one hour, as all the people there will trying to buy at that same time.

P: It was difficult. We need to go and stand in the line for an hour by maintaining social distancing. Some shops were open after one hour also.

I: If at all you wanted to buy products from a far place. Were you allowed to travel by bus for getting those products?

P: Where were the buses? We need to go through thookanam only….

I: How did you go… Were the shops open?

P: shops were open only for limited time. We need to walk for only ten minutes, but the waiting time was very lengthy. If we go early in the morning then we can return back before 12 after getting all the products.

I: Was the food products enough for you?

P: It was not available, but we managed with whatever we had. We managed with greens. We made sambar and had using the toor dal given in the ration.

I: Were there any difference in the price of food products before and after this COVID pandemic?

P: Price was high only…for one day…

I: Can you give example on what and all were priced higher?

P: Onion was around 100 rs and tomatoes were 60 rs per kg. we used to buy things for gravy for aroung 200 hundred rs usually, but during that time we sent only around 50 or 100 rs.

I: You have reduced your quantity to half…

P: Yes

I: Apart from this what other challenges did you face during COVID?

P: eating was itself a big concern. We used to make ragi or rice porridge. Children were not going to school, so they will have difficulty in buying things and eating. Everyday they will tell that they want to buy something and eat. But at that time there was financial crisis. Children will cry. We will make something out of tamarind.

I: Were you facing financial crisis at this time? You borrow money inorder to meet your daily expenses right?

P: yes. Only me and my brothers are the earning members in the family. During the lockdown we managed with the borrowed amount only.

I: Ok. During lockdown time, how helpful was the government to you?

P: government only provided us with rice and toor dal. Apart from that they gave rs. 2000 per ration card. We ate with that only.

I: Essential like milk, rice, did you get that?

P: We did not get anything. We were in such a difficult situation. That too with kids it was very very difficult. Kids were crying for milk, coffee….

I: So how did you try to manage?

P: By consoling them…there was one COVID case in our neighbouhood, so children didn’t want to out go out and they calmed down.

I: Everything is fine. Whatever you told was very useful for us. Do you like to ask something to me….regarding the food items which we provide or do you wish to share anything else?

P: Nothing else. With putting all your efforts, you have come here to enquire about our health, that itself is enough for us.

I: Good thing mam. Whatever you shared with us was very helpful to us. We will recommend whatever you said to us for sure. Thank you.

# **Individual Interview: HHC-F-7**

I: Okay let's move the next topic. We have been  in covid pandemic for the past two years and it has been problematic. It was a huge problem in the beginning when it started in March 2019. During the first six months of it starting there were several lockdowns. what kind of difficulties did you face during those times?

P: It was very problematic. There were no stores open. If they go to work we would only get rice. For rest of the food supplies we have to go to ration shops. We would get only limited things. It was very difficult and risky. We did not have much income so we could not buy much and store it.

I: Were they going to work from your home at that time?

P: they used to go for small household chores taking risk and to drive car at KV tex.

I: okay okay.

P: On the way there were police. We had to give them money. If they go to work at 6 in the morning, it will be 12 in the night by the time they return. I was risky to go. We did not have the money to buy food supplies and store it.

I: okay okay.  How was your income affected during those times?

P:  It was very less. Only if he could go for work would we get the money. The monthly salary that we get has to be used for the whole month. We would fear when they go to work, whether they have eaten it or not. We would wait for their return from work because  only they would get the food. Mother in law used to go to work but she does not get paid regularly. It was very difficult at that point.

I: There was unemployment?

P: yes sir. We were at home because there was no work.

I: okay.

P: During those times, we had to manage with the food supplies that were distributed. It was very difficult.

I: Did you have to borrow money or get loan?

P: yes we borrowed a lot.

I: okay. Have you got rid of the debt or are you still paying.

P: we are still paying.

I: how much money have you borrowed till date.

P: almost 25k.

I:During those difficult times, did you reduce the food intake?

P: Even if we reduce the food intake we won't reduce much.

I: Okay. During the lockdown were the shops open for you to buy food supplies?

P: Only the small shops in the streets would be open. The main shops were not open. They used to open the shops only at certain times. The shops inside the streets were open so that was useful. if we wanted milk or tea immediately it was helpful to get from there.

I: did you get all the things you wanted?

P: some things were available. whatever was available we managed to cook with that.

I: Were the rates of the things hiked?

P: yes it was hiked a lot. Not many had jobs or income at that point. Everything was very expensive.

I : so you somehow managed at that time.

P: yes we managed.

I: Okay thanks a lot for sparing your time for us.

# **Individual Interview: HHC-F-8**

**Interviewer: [**49.51] Well, we have asked all the questions related to food for so long, that all is over, now we are going to ask you some questions about covid. To get rid of this, should we put a lockdown in that period 2020 or not?

**PARTICIPANT:** It was a situation where we had to go out for a day because of the lockdown that we had been here for a quarter of a month, so none of us could go to work. Not only that, we had a lot of financial problems and we had a lot of trouble coming there without any income.

**Interviewer:** Do you think how much the income has been affected during the covid period?

**PARTICIPANT:** During the corona period, our income stopped in a complete way, we came and took loans from the affected neighborhood, we didn't come to buy milk, we bought food products like this, we came to use it like this. We brought a 6,000 or 7,000 like that. We. Because of this money crisis, we have been coming and going to manage that we have taken loans from everywhere in the neighborhood, except for food items, we have not come and spent money.

**Interviewer:** During the normal time and in this period, you are struggling with what kind of things, you are thinking like that?

**PARTICIPANT:** [52.43] Now, during the Corona period, we use to suffer a lot and it was very difficult and at the same time, it was difficult for us to travel somewhere outside and not work.

Let it be a matter of food, let it be a matter of going out to buy things. At that time when the shops were open, father was not feeling well, so we use to buy things after finishing the housework and got up in the morning to do it, while we should come and buy the things available at that time. It was very difficult at that time. It was very difficult for us to go out to buy any kind of food, whether it was to buy vegetables or not.

If we want to go to the shop, or to buy some things we should go on a bicycle. When the police came, it was very difficult for us.

**Interviewer:** How much you suffered for buying things and for others.

**PARTICIPANT:** During covid period, when mother comes and goes to work, she comes and brings spinach, moringa leaves and all that, and then some greens. I have prepared the eggplant and come and give some fries. If not, come at home and bring something. If it’s not enough? Then, we use to break it up with your hands of wheat or rice items. If not, make porridge for a few days. This is how we managed to come at that time anyway.

**Interviewer: [54.20] T**hank you so much for taking so much time to answer all our questions calmly and patiently. Thank you very much.

# **Individual Interview: HHC-F-9**

- **Interviewer:** Okay. Coming now, the corona section, a few questions, what this corona period has come, isn't it? Shouldn't we put a lockdown all this time? On that note, a few questions?

**Participant:** Let this corona come in time in our 2020 and put all the lockdown or not?

- **Interviewer:** Did you get enough stuff then?

**Participant:** No, nothing is available. I was just sitting at home. All of us were sitting at home looking at the sign. Nothing is available. The shop will not open. Everything should be at home.

- **Interviewer:** [48.38] Now, during those corona periods, no one had a job. How did you manage to overcome them, home?

**Participant:** It was very difficult to stay simply at home. There is a small shop nearby our house. We use to go and get things from to them, take a loan and spend like that.

- **Interviewer:** At that time, how did you buy it?

**Participant:** We bought it from some of the known persons

- **Interviewer:** How much have you bought?

**Participant:** Don't know that. They won't tell me about it. At home, only they know. Okay.

- **Interviewer:** Come now, in these corona times, to eat food, our food is very important for us. Did you come and not eat because there was no food available?

**Participant:**  we were. Rather than not being available, the rate should have been higher, first. This is the time that we cannot afford, saying so and so, all the grocers calculated were there to look at us that we could be pay or not. That's why

- **Interviewer:** [49.44] Without eating anything to buy it, then, beyond this, how many things have you managed, during Corona period?

**Participant:** During the Corona period, eating will be difficult. From here, even if we don't go outside, it will be difficult. If we have a function, it was difficult. From here, we can't go anywhere.

- **Interviewer:** Okay. I told you have taken the loans during the corona period. Have you covered it all now?

**Participant:** Don't know that. My parents will not tell us that i don't like it if they take a loan.

- **Interviewer:** [50.11] During the corona period, the shops were closed and it was very few only, didn’t? So, the food item, come now, is the food available or no at that time. How do you buy? How did you manage?

**Participant:** Available, but I have to go a long way to buy it. Just go to the store and buy it. That's it, keeps takes some time.

- **Interviewer:** [50.29] How come you managed?

**Participant:** For all it takes a lot of time, we are not the ones who can't eat time, the time they to buy things at far distance shops and we have to maintain a queue for social distance, and it’s very difficult for us.

- **Interviewer:** Okay.

**Participant:** So, maybe even the food should be cut, for sometimes.

- **Interviewer:** [50.36] In those corona times, Has there been a situation in your house without food? How long has it been?

**Participant:** Some time we have only one time to have. Anyway, let's eat for three times a day. Sometimes we don't eat. Time is, of course.

- **Interviewer:** [Okay, all the things you told me today and were very useful. Okay. Thank you very much for spending an hour for us.

# **Individual Interview: HHC-F-10**

- INTERVIEWER: [31.23] Till now we have discussed about the quality of items and its usage and the advantages and disadvantages of getting the coupon. Here after we are going to discuss about the Covid lockdown and the difficulties and sufferings that who have faced at those critical times?

During those lock down times mostly during March -April 2020have you find any difficulties for going job or any other related work ?

PARTICIPANT: Absolutely we have suffered a lot sir. Since, there is lock down, we couldn’t go for work we couldn’t have enough money to eat properly because of poor income. Schools are also remains closed and everyone get stuck at home and we couldn’t afford for buying the basic and essential amenities. So, we have adjusted with some of the available items.

- INTERVIEWER: [33.29] So is your income reduced a lot?

PARTICIPANT: No sir we don’t have any income. We are just prohibited from going to any job. We have adjusted with some of the items available which we previously brought already.

- INTERVIEWER: [33.38] Since you are jobless and you need to spend money for that, have you borrowed any money from any of your friends or relatives?

PARTICIPANT: Yes sir. Since we are prohibited from going to job, in order to maintain the food and other expenses we have borrowed some money at those times.

- INTERVIEWER: [34.03] So you are telling that you have borrowed but yet then also, you have adjusted the money so much to maintain the expenses. Is it right?

PARTICIPANT: Yes sir. If we borrow some 500 rupees, we have to adjust it for two weeks of time. Since, at those times every grocery items were available at more cost we couldn’t buy healthier items like milk, meat, fish and other things.

- INTERVIEWER: [ 35.32] Since you have adjusted so much of the money, how much you have borrowed and adjusted?

PARTICIPANT: If suppose we use to borrow 1000 rupees generally but we use to spend only 500 towards it and we have saved at least some 300 for the next week.

- INTERVIEWER: [35.44] After passing the lock down time have you seen any differences between the two various life style?

PARTICIPANT: Before Covid lockdown timings we use to cook at least twice for buying non-vegetarian items and during lock down we couldn’t buy not even any single time. We just adjust with available one or two vegetables at home.

- INTERVIEWER: [36.50] During the lockdown time most of the shops were closed. How did you manage at that time?

PARTICIPANT: Yes, sir certainly we have suffered a lot since most of the shops getting closed at those times.

- INTERVIEWER: [37.25] Is the shops in your area get closed at those times?

PARTICIPANT: Yes sir. We have also most of the shops wee closed at those times. But very few shops only opened. In those shops also, only a particular time only some shops were opened. At those time we have to buy things in a very long queue. Since, it was lock down people use to buy groceries and other things for whole month needful. So, most of the things were unavailable because of scarcity. We couldn’t buy like those since we don’t have enough money to get it.

- INTERVIEWER: [37.46] During the lock down timing in urban areas most of the things were available at the departmental store ,likewise is there any small shops were available at your rural side?

PARTICIPANT: If they are announcing the previous day we couldn’t go and get the needy things suddenly and if suppose we are go we couldn’t get all available things since there will be a long queue in getting the things. So, most of the thing were sold out at those timings. Thats how we have suffered a lot.

- INTERVIEWER: [38.27] Is that all grocery items were available at that time?

PARTICIPANT: No sir, most of the things were unavailable and we couldn’t buy all the things.

- INTERVIEWER: [38.42] As you said most of the things were unavailable. How you have faced those situations?

PARTICIPANT: We have managed somehow with the available things. But we couldn’t buy and get healthier items like Fish, Meat, etc…We use to have often green leafy vegetables, using Raggi we use to make Roti, Kanji and Anganwadis health mix, Koozh like items, and sometimes we have taken curd etc. This how we have managed since we couldn’t go for any job and we couldn’t buy any healthier items.

- INTERVIEWER: [39.50] So you have managed with Curd and other least available items at your home, Isn’t it Right?

PARTICIPANT: Yes sir, we couldn’t have any healthier food items. Since, we have not afford for it. This is how we have suffered a lot.

- INTERVIEWER: [40.04] Have you got any of the food and other things received through any of the Government side during the Covid lockdown times?

PARTICIPANT: Yes sir, some of the political parties use to give some amount of rice, vegetables and some of them provided food also. We are in need to get those items.

- INTERVIEWER: [40.21] Did they give quality of food items?

PARTICIPANT: Somehow, it was good quality only sir. We don’t have money do buy anything and we couldn’t go to any job, so we are in necessity to buy those things for our survival needs.

- INTERVIEWER: [40.39] Till now I have asked you so many questions and you have patiently answered well for those questions. If you want to ask anything you can ask?

PARTICIPANT: The given food items were so good and its very helpful for us. You have answered so many questions to me. If you give nutritious food items like Cashew, Badam, Dates and Egg, Horlicks it will be so helpful for us to get healthier.

- INTERVIEWER: [41.41] Okay mam, surely, we will convey your suggestion to our higher officials. Thank you so much for your valuable time spent for us mam. Thank you.

# **Individual Interview: HHC-F-11**

- INTERVIEWER: Well said mam. During the lockdown period of the pandemic,Everyone had to be at home and did suffer because of it. During that period of time, how did you manage at your home?

PARTICIPANT : We faced a lot of difficulties and suffered during that time period. We suffered a lot of things. My parents couldn’t go to work, no one could go outside. Since there was no job, we were stuck a lot for getting anything. Since we were not allowed to go outside we couldn’t buy the essential necessities. We have borrowed some amount of money from outside. We couldn’t return it soon.

- INTERVIEWER:[32.38] You said your parents were not going for a job, and you borrowed some money from outside. How much money have you borrowed ?

PARTICIPANT : For a month they borrowed around 5000-6000 to overcome the expenses.

- INTERVIEWER: [32.45] Have they returned the borrowed money?

PARTICIPANT : I am not aware of that sir. Only my parents know about that sir.

- INTERVIEWER: [32.51] During the lockdown time, how did you get the grocery items for your family?

PARTICIPANT : We faced a lot of problems getting things from outside sir. Most of the shops were closed and we had to get it from a faraway shop. In those shops also we had to get in after standing in a long queue. Sometimes we did not get enough things because of less money. So, we have adjusted a lot, mainly for food.

- INTERVIEWER: [33.07] How long have you traveled to get these groceries?

PARTICIPANT : We had to travel to Thiruvennainalor which is 3-4 kilometers from here sir.

- INTERVIEWER: [33.14] How did you get there ?

PARTICIPANT : We used to go by my father’s bike sir.

- INTERVIEWER: [33.16] So did the petrol charges also become a problem ?

PARTICIPANT : Yes sir. Of course.

- INTERVIEWER: How did you manage these things during those times?

PARTICIPANT : We have suffered a lot during that time. We had to spend a whole day getting these things. Moreover we couldn’t get all the items. So repeatedly we had to go there and get it. We have suffered a lot like that.

- INTERVIEWER: [33.59] Have you consumed meals all three times during the lockdown period?

PARTICIPANT : No sir, we had only 2 times, since we couldn’t have it all three times. Also we couldn’t get milk either. It was very rare to get it.

- INTERVIEWER: [34.14] You didn’t have it because of unavailability or because of other reasons?

PARTICIPANT : Since all were stuck at home, we couldn’t afford everyone's needs. So, we had meals only twice.

- INTERVIEWER: [34.20] So you have suffered?

PARTICIPANT : Yes sir. We have suffered a lot.

- INTERVIEWER: [34.32] At that time, some of them received some help from outside. Did you receive it?

PARTICIPANT : No sir. We never received anything.

- INTERVIEWER: [34.59] Did you and your family members get depressed at those times?

PARTICIPANT : Yes sir, we were depressed a lot. Since we did not have enough money to fulfill our daily food needs and other requirements. We couldn't go outside for jobs and other things. We were stuck inside. We couldn’t afford to get our educational needssir.

- INTERVIEWER: [34.57] What were the difficulties you have faced personally with regard to your education ?

PARTICIPANT : Since it was online, they had given so many assignments to us. We couldn’t do it since we lacked money to recharge our internet pack and so many other things.

- INTERVIEWER: [35.07] How did you overcome those challenges at that time?

PARTICIPANT : We were able to overcome those critical times by borrowing money from outside sir.

- INTERVIEWER: [35.18] Have you returned all those debts?

PARTICIPANT : Somehow we have returned it sir.

- INTERVIEWER: [35.22] So at that time you have adjusted within your family. Isn’t it ?

PARTICIPANT : Yes sir. We have adjusted a lot. We have shared food meant for 5 people among 8 of us. We have suffered a lot like that.

- INTERVIEWER: [35.37] So did you lack consumption of healthy food items at that time ?

PARTICIPANT : Yes sir. We couldn’t afford healthy items. We had normal food only.

- INTERVIEWER: [35.39] Did you get the normal day to day requirements at that time?

PARTICIPANT: No sir we couldn’t get it. We couldn’t get oil, soap and mostly milk was unavailable at that time. We used to go far away to get the milk.

- INTERVIEWER: [36.04] So you have faced such kind of situations.

PARTICIPANT : Yes sir. There is scarcity of daily wagers to meet our farming needs. Since we had our online classes we had gone for that. My father struggled a lot to pay for fertilizers for our land.

- INTERVIEWER: [36.37] Who are all working on your farm land?

PARTICIPANT : My parents used to go for the farming work. Father will take care of the agricultural needs and my mother will go for some farming work like removal of weeds from land and other kinds of things.

- INTERVIEWER: [36.50] During the lockdown time, did any of your land products help you overcome your financial needs?

PARTICIPANT : No sir. At that time only we have started planting our crops. We couldn’t afford to meet our financial needs at those times.

- INTERVIEWER: [37.00] Were you able to harvest any vegetables from your farm land?

PARTICIPANT : No sir. We didn’t get anything. Everything we had was bought from the shops only sir.

- INTERVIEWER: [37.04] Did you face any difficulty in attending your online classes?

PARTICIPANT : Yes sir. The way of teaching through online was not clear and we have faced some trouble in that. We couldn’t handle it. We could not go to college and somehow we have managed sir.

- INTERVIEWER:: [37.23] What do you mean by that , Can you explain it clearly.?

PARTICIPANT : The mode of teaching was not clear sir.

- INTERVIEWER: [37.44] During the lockdown your parents were staying at your home. Did they get depressed ?

PARTICIPANT : Since we couldn’t afford our daily needs they were depressed a lot and therefore they suffered.

- INTERVIEWER: [37.58] Have they told you about this anytime?

PARTICIPANT : Yes sir. They used to speak about this to me many times. They said the Government could have helped in some way to overcome this situation.

- INTERVIEWER: [38.06] Were there kids around at that time?

PARTICIPANT : Yes sir. My sister’s kids were around at that time. Even to get milk for the kid we had to go far away. Moreover, they had increased the cost of milk also and there was scarcity of milk. We couldn’t have milk at that time sir.

- INTERVIEWER: [38.13] Since you are living in the village, have you received the Government free supplies provided at those times?

PARTICIPANT : We have received only the items given through ration shops and not from any other place. Even those supplies some of them didn’t receive.

- INTERVIEWER: [38.49] How do you feel now when compared to that situation?

PARTICIPANT : Since my parents are going for a job now they are running the family smoothly.

- INTERVIEWER: [39.04] How do you feel about your health and education right now?

PARTICIPANT : Everything is fine sir.

- INTERVIEWER: [39.06] Thank you so much. You have patiently replied to our questions in a detailed and calm manner. You should eat properly and have to take care of your health and your family members' health. Thank you so much.

# **Individual Interview: HHC-F-12**

- INTERVIEWER: [37.15] Till now we have discussed a lot of things about the food items, their quality and mode of receiving and so on. Next, we are going to ask some questions about the problems and different challenges and difficulties you have faced during the hectic COVID period of timings?

For us the COVID lockdown started exactly from the month of March 2020 to June 2020.How come you are affected during the lockdown period?

PARTICIPANT: Since we own a grocery shop, we use to get things needed for our daily routine use. So, we are not much affected in this type of issues.

- INTERVIEWER: [38.03] Have you managed till the whole period of lock down time from your shop?

PARTICIPANT: Somehow, we managed using our shop. After that we use to get the grocery and other essential things through online shopping only.

- INTERVIEWER: [38.40] What about the Family income during that period? Is it affected during those times?

PARTICIPANT: Absolutely our income has affected during that period.

- INTERVIEWER: [38.50] For to manage the expenses, have you brought any money towards your expenses from any one of your friends or relatives?

PARTICIPANT: No mam, somehow, we have managed.

- INTERVIEWER: [38.59] Because of that hectic situation have you reduce your money spending towards food and other cooking needful?

PARTICIPANT: No mam, we never use to reduce the money towards it.

- INTERVIEWER: [39.32] As you already said for a weekly wise you are spending some thousand rupees for other nuts fruits and other things. Is it same during the Covid lock down time?

PARTICIPANT: Yes, it remains the same for those time.

- INTERVIEWER: [39.45] Is it the same after Covid time also and now?

PARTICIPANT: Yes, it is same now and after covid time also.

- INTERVIEWER: [39.51] You would have followed certain schedule for maintaining expenses before the Covid period. Is that causes any changes before and after Covid lockdown timing?

PARTICIPANT: We never maintain such kind of schedule for maintaining the expenses. It remains the same for all time.

- INTERVIEWER: [40.10] Have you faced any difficulties in buying food and other items during the lock down time?

PARTICIPANT: Are you asking in terms of financial or in the way of getting things regarding this?

- INTERVIEWER: [40.31] Incase of getting the food and other items?

PARTICIPANT: Yes, we have faced a lot of difficulties since most of the shops get closed, so many items are un available and the time restriction all these kind of things causes lot of trouble at those times.

- INTERVIEWER: [40.51] Have you gone to and get the necessary things at that time? Is, the shops were closed or open?

PARTICIPANT: We have gone at the specific time and we use to get the things.

- INTERVIEWER: [41.09] Is all the essential items are available at that time?

PARTICIPANT: We use to get some limited number of items. So many items were unavailable during the lock down time. Since, we own the shop we had taken some things from the shop. This is how we manage to adjust the things at those time.

- INTERVIEWER: [41.32] How you managed to adjust the unavailable things?

PARTICIPANT: We have taken some of the available items from our shops other than that we have adjusted in using them.

- INTERVIEWER: [42.08] During that lockdown time have you use to get things from any Ration shops?

PARTICIPANT: We never used to get anything during those times. But, from the

Government side we received some groceries 3 to 4 times. So, we use to have used these things.

- INTERVIEWER: [42.40] what are all the things you have received at that time?

PARTICIPANT: We have received some amount of Rice, Dal items and Vegetables.

- INTERVIEWER: [42.47] Till now, we have discussed a lot of things regarding our food items, their storage, quality, quantity up to the Covid time. You have patiently replied our questions. If you want to tell or ask anything you may tell us. Thank you so much.

PARTICIPANT: The food items that you have provided helps us in getting improvement of our health. It was helpful for us very much. We all consumed everything. Apart from the given food items you can give dry fruits, nuts and other healthier items. It can help us to improve some more. Instead of giving, groundnut in excess you could have given White Channa dal since it was not fit for my intake. Otherwise, all items helped us so much. We all consumed it fully.

Thank you so much….

# **Individual Interview: HHC-M-1**

I: okay. During the corona pandemic, there was lockdown, how much did it affect your income?

P: we have suffered a lot I the last one year. Currently we are in rented home. We are not able to pay rent. There is no facility for water. We have to buy water as well, how could we pay for food supplies. This was very difficult for us.

I: before covid what was your income? After lockdown how much did your income reduce?

P: I used to earn 15000 per month. That was the entire income. People at home know this and also the people I have borrowed money from know this. After lockwdown there was not even income if 5 paise. I did not go to work , I was not able to go to work. It was very difficult.

I: the how did you manage during the lockdown?

P: during the lockdown we had rice from ration store. We ate the rice given by government. The rice given by government was very useful.

I: you have faceda lot of diifculty. Have you borrowed money and bought food supplies? Was there situation where there was no cash in hand?

P: it was difficult. When borrowing money they refused to give. I had to plead to get the money. We cannot blame anyone for it, covid affected all of them.

I: How did you reduce your expense during the covid period?

P: we ate only once in a day instead of three meals. We had only once a day with that money.

I:were you able to get the food supplies from the shop during the pandemic.

P: we did not get any food supplies, there were no shops .

I: did you get the essential food supplies from the government?

P: no we did not give anything. They gave only milk packets for children.

I: were the shops open during the lockdown to buy food supplies.

P: no nothing was open.

I: thanks a lot sir.

# **Individual Interview: HHC-M-2**

I: last two questions.. how was your income affected during the lockdown period?

P: There was no income during lockdown period. No income for 6 months. Nothing was running properly. The police men used to beat if we come out of home. It was worst but now there are no restrictions.

I: During lockdown you didn’t go to work for six months, How did you manage?

P: during lockdown, only to certain extent people were controlled. Later on they were not controlled. People were going outside. We can afford to eat only if we work.

I: What was your income before lockdown? How much of your income reduced before lockdown?

P: Before lockdown I had Rs 15000. During lockdown I had nothing. I used to get half of it around 7000.

I: During that period did you borrow money from others to get food supplies for household purposes?

P: I used to get money from others. I may also give money to others. Without that the family cannot run. This happens in every home. Like this we have borrowed and also given to others.

I: Because of lack of income, did you reduce quantity of the food you ate?

P: We could only eat what we had at that time. If we had lot of money, we can eat whatever we want. If there is shortage of money at home, we can only eat what we have. We cannot borrow more because of that. We eat non-veg food sometimes and veg food the rest of the time. We had to eat simple food. There is no hard and fast rule for eating this or that. We had to change oursekves depending on time and situation.

I: Were you able to buy the food supplies during lockdown?

P: Hmmm, we got it. Not in the main area but in small shops.

I: During lockdown did you had trouble buying food supplies?

P: hmmm..there was difficulty.

I: What type of difficulties did you have?

P: We could not go to the main road to buy things. But the hidden stores sell things at high cost. We bought 10rs worth product at 13rs because there were no other shops so they sold at high cost. We were compelled to buy.

I: So you were in a situation of buying things at high price?

P: yes.

I: how do you manage this?

P: We can only get a little amount as debt saying we will return in a week. We will buy only what we need.

I: Did you get food supplies? Or were you unable to go to store?

P: I was unable to go to the stores. But there was a situation where everything needed to be adjusted for the family.

I: What kind of shops were closed and what were open?

P: All shops were closed.

I: Then how did you buy the food items?

P: the food items were given at place where there were no police or else the police will seal the shop.

I: Have you seen the stores getting sealed?

P: hmm..many stores were sealed. This happened at the initial stage of lockdown.

I: during that time, did you get enough amount of food items?

P: no..they raised the amount of each product and sold. They stored and sold it.

I: Did you feel that the price of the milk may raise? For example the value of milk packet of 10rs would have raised?

P: It is an essential item for everyday usage. Some people cannot be without drinking tea so they need to purchase. So, milk, tea powder, sugar are daily usable items and need to be purchased.

I: Thank you so much. We spoke for an hour. We make sure we apply your suggestions and opinions.

P: thanks.

# **Individual Interview: HHC-M-3**

**INTERVIEWER: [42.10**]  Ok Let's Move To The Next Question Coronavirus Started From March 2020 It Was New For The Whole World Now I Will We Asking Some Questions Regarding It. When It Started In March 2020 There Was Extensive Lockdown Nationwide What Was The Problems Faced By Your Family At That Time

**PARTICIPANT**: It Was Very Difficult For Us. We Can Go Out Anywhere Even To Get Essentials And We Were Also Scared Of Contracting Coronavirus From Outside

**INTERVIEWER: [43.05]**  So During The Lock Down Nobody Was Able To Go Outside Their House When You Were Not Able To Go To Work How Was The Economic Situation Of Your Family?

**PARTICIPANT**: When I Was Not Able To Go To Work I Did Not Have Any Money We Have To Borrow Money To Spend On Food This Was How Those 6 To 7 Months Of Lock Down Were.

**INTERVIEWER: [43.35]** Ok During The Intense Lockdown Did You Have To Borrow Money?

**PARTICIPANT**: People Were Hesitant To Even Lend Money Then

**INTERVIEWER: [43.39]** Did You Borrow Any Money During That Time?

**PARTICIPANT**: Yes Sir Like 1000 Of 2000 From Someone We Know To Spend On Food

**INTERVIEWER: [43.50**] Because Of Situation Like That Were You Forced To Reduce The Food Requirements Of Your Family?

**PARTICIPANT**: Definitely Sir Compared To The Usual Food We Take We Reduce The Food Intake During The Lockdown Rice Or Whatever It Might Be We Reduce The Amount We Buy Usually And Used It Very Carefully.

**INTERVIEWER: [44.22] Were** You Able To Have Three Meals Per Day Or Did You Have To Reduce That To?

**PARTICIPANT**: We Had Three Meals Per Day But We Reduce The Quantity We Take Per Meal

**INTERVIEWER: [44.45]** So You Had No Earning, You Had To Borrow Money And Bring Down Your Food Intake How Long Did This Last?

**PARTICIPANT**: It Lasted For 6 To 7 Months Until The Lockdown Was Lifted Then After That Everything Started To Go Back To Normal Slowly.

**INTERVIEWER: [45.15]** How Long Did You Have To Work To Pay Back The Money Borrowed During Lockdown

**PARTICIPANT**: We Have To Mortgage Whatever We Had And Pay Back The Debts Then We Slowly Paid For The Mortgage

**INTERVIEWER: [45.48**] During Lockdown Shops Were All Closed What Did You Do For Essentials, Did The Government Help You In Anyway?

**PARTICIPANT**: Some Charitable Trust Provided Rice And Other Things And Government Also Gives Some Amount 1000 Rupees And Free Rations

**INTERVIEWER:** [46.17] Even Though Free Rations Were Given How Did You Manage To Buy The Other Food Requirements?

**PARTICIPANT**: The Bought Them From The Savings That We Had

**INTERVIEWER: [46.55]** Did You Have To Spend Too Much For This Food Items?

**PARTICIPANT**: Yes Sir Usually Oil Packets Are Sold For 95 Rupees But During The Lockdown Time It Was Sold For 125 Rupees So We Have To Spend More Than Usual

**INTERVIEWER: [47.35]** Was There A Time When You Were Not Able To Get Some Essentials That You Need

**PARTICIPANT**: Yes I Like Garlic, And Trimmer Blade All Were Not Available

**INTERVIEWER: [48.02]** Did The Lock Don't Have Any Other Impact On You?

**PARTICIPANT**: I Was Not Able To Go Out And Work We Had To Stay At Home All The Time

**INTERVIEWER: [48.27**] During Times Like That How To Do Keep Yourself Refreshed

**PARTICIPANT**: There Was A Small Waterfalls In The Neighborhood With Lot Of Trees I Used To Go There To Take Bath And Spend Some Time There

**INTERVIEWER: [48.58]** Thank You For Sharing All These Informations With Us.

# **Individual Interview: HHC-M-4**

I: okay the next question is, the pandemic had started on since March 2019. There are many issues and problems were going on till now for all. So I am going to ask few questions regarding this. What are the difficulties you have faced during the first lockdown in the march 2019?

P: we suffered only for food because my father and mother had no jobs at that time. We were not able to take nutritious food, but ate just for hunger. We don’t have sufficient money to buy those healthy food items.

I: okay.  How many days do your salary got affected at the beginning?

P: we got affected lot. Because both my father and mother have no jobs. That was so painful. We were not able to eat.

I: okay. During the difficulties, did you get any loans or debts? Have you gotten any loans using the gold jewelry?

P: yes during lockdown times, we used my mother jewels for loans. We bought food using that money.

I: have you taken back the jewels from bank?

P: no. half of the jewels were in bank.

I: so the effect is being the same from the beginning?

P: yes.

I: due to those situations were you forced to eat less amount of food and change your lifestyle because of that?

P: not the less amount of food, but we used to eat only twice in a day. We reduced the number of times we eat in a day. Since we have no money we done like this and saved some amount of things or money. We focused mainly on food.

I: okay. During the same lockdown times, have you faced any other difficulties to buy things from nearby shops?

P: yes we couldn’t able to go outside during the lockdown. If it even opened we have no money to buy things.

I: okay. Does the shops opened all the time during pandemic?

P: no we have time restrictions. The shops were opened sometimes upto 12pm. Since we have no money, we bought only fewer things. For one week we buy things, and then the next week we won’t have money so we are not able buy.

I: do you get any help from the government side like giving any food items or money?

P: yes we got. But it was not that much useful. It was useful only for two weeks or one week.

I: have you faced any problem while getting the things from shops?

P: yes. The thing is we should go on time to buy the things. We have to buy the things within the budget. During normal times, we have money. So we bought the things as our wish. But we are not supposed to do the same at the pandemic times. We also don’t have sufficient time.

I: Do all the things were available in the shops during the pandemic times.

P: no not at all. We are able to buy only vegetables and not more than that.no other things were available. So we couldn’t buy.

I: okay. What type of things you bought during that times and what type of food items you bought?

What type of foods you prefer to eat?

P: we buy vegetables and basic needs like paste, brush and cooking items like oil, chili powder. We don’t get any unwanted items.

I: so you have struggled a lot in this pandemic situation?

P: yes.

I: that effect is still continuing?

P: yes. Still we are not able to take the jewels from the bank.

I: okay okay. Till now you have shared lot of things with us. All was good. We believe it would be useful further. Thank you sir.

P: okay sir.

# **Individual Interview: HHC-M-5**

I: Lastly, during the COVID period how did u manage and how did it affect you? So COVID started in March 2020 how it affect your life (42.21)

P: It was very difficult during that time, we used our father’s savings and we had some land we harvested rice and we managed with few vegetables and due to restriction it was difficult to sell vegetables

I: I did not understand. you used to sell vegetables for living?

P: My father used to see groundnut and this time he harvested brinjal and groundnuts. During covid time he couldn’t sell

I: how much your produce or income got affected due to covid? How much was your income during that time?

P: Every time we used to get 15,000 this time we got around 10,000

I: You said during covid times your income was affected were you able to purchase food items during that time? (43.46)

P: No, we were able to manage in our farm we had cultivated few crops required for home too we used that

I: what all did you harvest

P: Brinjal, Tomato

I: Did you reduce the quantity of food you ate

P: No, we consumed the usual amount of food

I: Did u borrow any money during covid time for buying food items

P: No we did not (44.31) we used our savings and we managed with whatever was available

I: Where the shops open during that time

P: Departmental stores were open for 2 hours every day we used to go during that time to buy

I: were the shops crowded? how did u manage to buy

P: it was crowded but we maintained social distance

I: During covid time how did the government help you?(45.10) were u given ration?

P: they gave only rice at the ration shop, they did not give vegetables

I: You told only few shops were open

P: yes it was section 144 during that time so only few were open for limited hours

I: During the lockdown time was there any price hike

P: yes it was, during lockdown for their profits they increased the prices

I: can u say for example milk how much did they increase

P: They during that time the expenses were more

I: Were you able to buy daily essential items

P: yes

I: So there was no hindrance in buying items

P: No

I: Thank you for cooperating.

# **Individual Interview: HHC-M-6**

I: (28.02) 2 years back there was covid pandemic and how did you family manage during that time did u have any financial problems (28.23)

P: yes sir during that time there was no income we couldn’t find any work, we all were at home. There was no money to buy food I borrowed money to buy mobile also so we were in debts and we couldn’t hold mobile due so we had to borrow money to pay it even for buying food we borrowed money (28.51)

I: was there any problem to buy food

P: we had difficult because we dint have money to buy otherwise we could buy food from the shops

I: where did you borrow money from?

P: we got it from the landlord and few known persons

I: how much did you borrow?

P: Nearly 30,000 rupees

I: how much was the interest?

P: I don’t know how much was the interest but I was 10 paise interest

I: how did you repay it

P: I don’t know how. The loan is due

I: Ok, during covid lockdown time did you reduce the quantity of food that you used to eat (29.56)

P: they used to make food at home but there used to be a littlethat I  I was not going for work and we dint feel like eating, we did not reduce the quantity

I: during lockdown all the shops were closed in cities all the shops were closed and in villages few shops were open. Were there any shops open here were you able to buy groceries were all the essential groceries available?

P: here there were small grocery shops open all the shops on the main road closed there were few shop in our neighbours house those shops were all open

I: were you able to buy all that you wanted

P: yes, not all the items but small items were all available

I: were the shops open all the time (31.07)

P: no sir they were open for limited time only, they took care that the shop doesn’t get crowded. The items in the shop were also less because all the whole sale shops from where they brought were closed so many items were not there

I: were the prices high

P: no sir the prices were not increased. As far as the item was available they told it

I: due to lockdown were you able to go to shops

P: We did not go outside our village, we used to buy whatever was sold in our streets

I: ok because of it there was no much problem. But you went through hard times during covid. After covid did you start going to work again?

P: I was a student I was studying recently only I started working

I: OK, thankyou for giving your time and responding to my question. We will consider all your suggestions
