# Supplementary material for: Food insecurity in South Indian households with TB during COVID-19 lockdowns and the impact of nutritional interventions: A qualitative study
Source: PLOS Glob Public Health. 2025 Apr 9;5(4):e0004242. doi: 10.1371/journal.pgph.0004242 (PMC11981200; doi:10.1371/journal.pgph.0004242)
Supplement: S1 File — The COVID-19-related questions have been highlighted in yellow. (DOCX) [file pgph.0004242.s002.docx]

**Learning about Experience with Nutritional Supplementation (LENS)**

In-Depth Interview Questions - HHC

A. Experience with the intervention itself (type of food, quantity, etc.).

1. What do you think of the quality of food that is being provided to you? Please elaborate.

(**Prompts:** how is it comparable to Kirana shops, fair price shops? Are you happy with the containers in which the food is stored? Does it keep the food fresh?)

1. What do you think of the particular food you receive? Suggestions for improving the food items given to you?

(**Prompts:** Do you like the type of lentils? The type of rice? Are you happy with the amount of each food? Do the ingredients match the food you prepared in your home traditionally- Did you buy the same ingredients that are supplied to you before the intervention started?

1. Family members’ opinion on the food supplement?

(**Prompts:** Does your family think the food is good quality? Do your family members think that the amount of food is enough? Are your family members satisfied with the food? Do they think there’s a good variety of food?)

1. What other foods would you prefer? Please elaborate.

(**Prompts:** Suggest other ingredients: spices, fresh produce, snacks, etc.)

What other food items do you consume?

1. What ingredient(s) do you think should be discontinued, if any? Please elaborate.

(**Prompts:** Why? Would you prefer a different ingredient in its place?)

1. What is your opinion on receiving one ingredient as a food supplement?

(**Prompts:** Ex groundnuts, lentils, would you eat it every day? How would you eat it? Would you get bored with it? How long (weeks, month) do you think you would eat it for?)

1. How do you feel about eating a snack that is designed to be particularly nutritious? (**Prompts:** [maa ladoo, halwa, protein powder, Horlicks] How would you eat it? How frequently would you eat it? How long (weeks, months) would you eat it for?) –

Additional prompt- Do you think it is healthy to snack in between meals? Is it convenient for you to consume snack during the day. Is it allowed/acceptable at your workplace/school?

1. What is your opinion on the quantity of food that is given every two weeks? Among the foods that are delivered to you, which ones do you consumes completely? (**Prompts:** Which ones do you not eat? Why not? Is it that you don’t prepare the food, is it too much, do you not know recipes, you don’t like the kind of ingredients, you are bored with it, etc.?)
2. Are you buying additional food? What do you buy? How much do you spend on outside food per week?

(**Prompt:** Why are you buying additional food? Examples: not enough food, not enjoying the food, need more variety.)

How much do you spend on junk food and nutritious snacks per week?

1. Who prepares the food at home? Availability of time to prepare food with the given food supplements.

(**Prompts:** If you/they don’t cook, how do you get food? Do you prefer instant foods that are easy to cook?)-

How many meals do you consume in a day? How many meals you consume at home?

1. How often to do you eat meals outside of the home?

(**Prompts:** Why do you eat outside the home, where do you eat, what do you eat)

Do you prefer eating outside than at home?

1. Who consumes the food you are receiving as part of this program? Please list.

(**Prompts:** Understandable, when some people have extra food, they may be sharing with others. Have you had to give extra food to your relatives? Are you selling any food? If so, which food items? How much? Is it to pay off loans or for other reasons? Do you perceive the food to be unnecessary? Could you see others doing this?)

1. What effect, of any, do you feel the food supplementations had on your household? Please tells us about your experience with the food supplementation.

(**Prompts:** Any grievances or benefits? Ex economic, time, awareness about nutrition, etc.) Did the food supplements given change your diet plan? Did you have a fixed diet plan before the intervention?

In your opinion, do you think you have any unhealthy food habits ?

1. Are you consuming the multivitamin tablets which are being provided? Do you think they are effective and safe? If not, why do you think so?

(**Prompt:** How do you feel about taking the vitamins? Any benefits or problems?)

Do you think the multivitamin tablets affect your appetite?

1. What is your opinion on the role of these food items on your health and on the treatment of TB?

(**Prompt:** Did you feel like you were at a healthy weight when you started the intervention or did you think you needed to weigh more or less?

Do you feel healthier/like your health has changed since receiving this food? Do you think the food is useful or not for TB? Why?)

Do you fear gaining weight will make you unhealthy?

1. Is there anything more you would like to add?

**B. Delivery (better through PDS, as money)**

1. What do you think about frequency in which the food is delivered?

**(Prompts:** Should food be delivered more or less frequently? If so, why?)

2. What do you think of the way you receive the food supplement at your home?

**(Prompts:** Are you okay with someone showing up to your home to deliver food? Do you feel that food deliveries in any way bring attention to your family? Would it be ok if ASHA workers brought the food? Imagine that home delivery was NOT an option- would you like to pick the food up somewhere like Anganwadis or the TB clinic? Other ways?)

3. Are there other methods of receiving the nutritional intervention that you would be interested in? What are the drawbacks of the current method, if any?

**(Prompts:** Again, if home delivery was NOT an option, would you rather receive a coupon that you can use to go buy food elsewhere? Would you use the coupon for food alone? What would be advantages and challenges with food coupons?)

4.What are your views regarding cash transfers instead of receiving in-kind food?

(**Prompts:** Do you prefer cash transfer instead of in-kind food? Do you think you’d use all of the cash transfer for food or do you think you would use it to defray other household costs or loans? Do you have a bank account which allows you to receive money? Aadhar card? What would be the advantages and challenges with giving cash?

**C. Effect of COVID-19 pandemic on earnings and ability to obtain food**

1. Did the lockdown due to coronavirus disease affect your earnings in March, April, May and June 2020? If so, how much did your earning decrease? Did you have to take a loan? Did you have to decrease expenditure on food?

(**Prompt:** If you had to decrease the amount of money spent on food, how much did you have to decrease it? Was there a noticeable difference from before COVID?)

2. Did the lockdown affect your ability to buy food? Were you unable to go to the store to buy food? Were the shops closed? Did the shops have inadequate stocks of food?

(**Prompt:** If you weren’t easily able to access food, how did you compensate? Example: rationing)
