# Supplementary material for: Food insecurity in South Indian households with TB during COVID-19 lockdowns and the impact of nutritional interventions: A qualitative study
Source: PLOS Glob Public Health. 2025 Apr 9;5(4):e0004242. doi: 10.1371/journal.pgph.0004242 (PMC11981200; doi:10.1371/journal.pgph.0004242)
Supplement: S2 File — The COVID-19-related questions have been highlighted in yellow. (DOCX) [file pgph.0004242.s003.docx]

**LENS FGD Discussion Guide**

**Notes**

1. The FGD discussion questions do not need to be asked exactly as written. If a participant brings up an interesting point that you think is relevant, you can take a few minutes to discuss this topic with the group. However, if participants begin discussing subjects that are not relevant, it is important to return to the discussion questions.
2. When asking the discussion questions, try to focus on the *community* perspective. For example, you can ask participants to share their favorite food first, but you should especially focus on what foods they think *others in the community* would like best.

**Questions**

Composition of the meal

*We would now like to ask you about your experiences with the nutritional intervention.*

1. What foods did you like best of those provided? What foods did not you not like as much?
2. What did you think about the vitamins provided during the intervention? (Probes: when did you take the vitamins? Were there any problems with the vitamins? Did you see any benefits? Did you experience any adverse effects?)
3. Of the foods provided, which one would be most useful to other people in the community? Which one would be less useful?
4. What types of food would be nutritionally important for a TB patient? (Probes: high-protein, high fat, vitamins) What specific foods meet these requirements?
5. What are some new types of food that should be included in the food delivery? (Probes: Prepared meals? Snacks? Instant foods?)

Recipes

1. What are some different ways you prepared the food provided?
2. How can we help your community make better use of the food we provide?

Delivery methods

1. What did you think about the delivery of the food? (Probes: was the delivery convenient? Would another method be better?)
2. What delivery method do you think would be best for people in this community? (Probes: coupons, pick up at the hospital, pick up at anganwadi, pick up at ration shop, delivery by healthcare workers, delivery by ASHA workers)

Direct Cash transfers

1. Do you reliably receive monthly cash transfers for nutritional support? (Probes: Are the payments on time? Do you get them every month?)
2. How do you currently use the monthly direct benefit cash transfer provided by for the TB patient? How do you use the extra money you have when you do not need to purchase as much food due to this intervention?
3. What are your opinions on providing a cash transfer instead of food delivery? (Probes: would the money be used for food, or for other purposes? Would cash in hand or a bank transfer be better for people in this community?)

Stigma

1. What do your neighbors think about your food delivery? (Probes: do they ask questions? How do you answer them? Do you think the food delivery could lead to negative treatment?)
2. How can we prevent breaching your confidentiality when we deliver your food? How would picking up food versus having it delivered affect your treatment by the community?
3. Have you ever experienced any negative treatment due to the TB status of your household member? Have you heard of other TB patients in your community being treated poorly due to their TB status?

Problems/suggestions

1. What were some parts of the intervention that could have been better? (Probes: quality of the food, timing of the delivery, method of the delivery, amount of food.)
2. What changes could be made to improve the intervention in the future?
3. What would be the best method for sharing nutritional information, recipes, and advice? (Probes: phone calls, WhatsApp, in-person visits.)
4. What do you think about creating a community of families receiving food so you can share recipes and support each other using a mobile app? What would be some problems and benefits? (Probes: access to mobile phones, access to internet, level of interest.)

COVID-19 (Can integrate if on practice run, we have enough time)

*We would like to start by checking in about your experiences during the COVID-19 pandemic and the lockdown.*

1. How did the COVID-19 pandemic affect your household income? (Probes: did you work? Did others in your household work? Did you take out a loan or sell belongings?)
2. What changes, if any, did you make to your eating habits? (Probes: did you eat less food? Did you have to forego a type of food? Did you eat different types of food?)
3. What was it like to purchase food during this time? (Probes: were shops open as usual? Were products available? Were prices the same or higher?)
