# Supplementary material for: Food insecurity in South Indian households with TB during COVID-19 lockdowns and the impact of nutritional interventions: A qualitative study
Source: PLOS Glob Public Health. 2025 Apr 9;5(4):e0004242. doi: 10.1371/journal.pgph.0004242 (PMC11981200; doi:10.1371/journal.pgph.0004242)
Supplement: Attachment — Submitted filename: Response to reviewers 241024.docx [file pgph.0004242.s006.docx]

Journal Requirements:

1. **Please include a complete copy of PLOS’ questionnaire on inclusivity in global research in your revised manuscript. Our policy for research in this area aims to improve transparency in the reporting of research performed outside of researchers’ own country or community. The policy applies to researchers who have travelled to a different country to conduct research, research with Indigenous populations or their lands, and research on cultural artefacts. The questionnaire can also be requested at the journal’s discretion for any other submissions, even if these conditions are not met.  Please find more information on the policy and a link to download a blank copy of the questionnaire here:** [**https://journals.plos.org/globalpublichealth/s/best-practices-in-research-reporting**](https://journals.plos.org/globalpublichealth/s/best-practices-in-research-reporting)**. Please upload a completed version of your questionnaire as Supporting Information when you resubmit your manuscript.**

We have attached a complete copy of this questionnaire to the resubmission of the manuscript.

**2. In the online submission form, you indicated that "Data are available upon request.".**

**All PLOS journals now require all data underlying the findings described in their manuscript to be freely available to other researchers, either 
a. In a public repository, 
b. Within the manuscript itself, or 
c. Uploaded as supplementary information.**

**This policy applies to all data except where public deposition would breach compliance with the protocol approved by your research ethics board. If your data cannot be made publicly available for ethical or legal reasons (e.g., public availability would compromise patient privacy), please explain your reasons by return email and your exemption request will be escalated to the editor for approval. Your exemption request will be handled independently and will not hold up the peer review process, but will need to be resolved should your manuscript be accepted for publication. One of the Editorial team will then be in touch if there are any issues.**

We have included a copy of the COVID-19-related questions from the focus groups and in-depth interviews.

**Comments to the Author**

**Reviewer #1: At the outset, I must congratulate the authors and the research team for the work on TB - a very important public health hazard which is one of the leading causes of death all around the world, more so in India. The underlying causes like undernutrition and its impact are well studied and your study about food insecurity during the time of the pandemic definitely worth of notice.**

Thank you for your comments and your thoughtful review!

**General Suggestions:
1.In the earlier sections of the abstract, there is mention of TB LION and LENS without being referenced or paraphrased in the description which makes it difficult to follow. Is TB LENS the current study in question?**

Yes, the results presented in this paper are part of the TB LENS study, which is under the TB LION study. TB LION provides the nutritional intervention, and includes the quantitative and anthropometric/biological data collection. TB LENS focuses on qualitative interviewing and focus groups.

While reviewing the qualitative data from TB LENS, we realized that we had a plethora of data related to COVID-19 and food security, which did not directly apply to our main research questions for the TB LENS study. We decided to separate the COVID-19-related results into a separate manuscript so that we can give them the attention that they deserve. The remaining TB LENS results will be published in a separate manuscript.

Per your helpful suggestion, we have clarified the above and spelled out the acronyms, with the goal of increasing clarity as to how the studies are connected in lines 87-89.

*“Data were collected and analyzed as a component of the Tuberculosis - Learning about Experience with Nutritional Supplementation (TB LENS) study, a sub-study of the Tuberculosis - Learning the Impact of Nutrition* *(TB LION) study.”*

**2. I feel that the readers would need a description of what the pandemic lockdown was, how long it lasted, what were the existing sanctions for collection of food, water etc and the said events during your study.**

We have added some information about the pandemic lockdown, as remembered by local study staff who experienced the lockdown in Pondicherry in lines 126-132:

*“In our study region, the initial 21-day lockdown began on March 22, 2020. Residents had limited access to grocery stores; while some received groceries from NGOs or local politicians, many did not.  Typically, grocery stores did not open until 10 a.m. at the start of the lockdown, and were only open for half days later in the lockdown. The lockdown measures were strictly enforced by the police. The lockdown was extended through May 31, 2020. Thereafter, restrictions were lifted in a phased manner.”*

**3. We have been only provided with the "LENS questionnaire" used in the available data set for supporting documents which I don't think are completely relevant with the results you have presented as they cater only to two questions mentioned in the C part of the document for IDI. No mention of FGDs and how they were conducted!.**

We agree this was an oversight. We have now also attached the FGD guide in the supplementary materials. As the COVID questions were asked as part of the LENS study, they are embedded within the LENS interview guides. We have highlighted the relevant questions to make them easier for readers to find.

**4. The full and the short title title would need an edit and upgrade as its not very reflective of the study in question and there is no mention of the nutritional intervention and its impact as well during the pandemic as you have only selected household contacts of patients with TB if they have received the nutritional intervention!**

Thank you for your thoughtful critique. In response, we have updated the title to focus on the impact of COVID-19 on food insecurity in households with TB:

*“Food insecurity in South Indian households with TB during COVID-19 lockdowns and the impact of nutritional interventions: a qualitative study”*

**Specific Suggestions:
37-38: would suggest change to " driver for hastened disease progression or disease related morbidity and increased mortality during the "**

Thank you, we have made that change.

**47-48: Since the intervention is not part of the study being described, featuring it as part of the results of the study seems and leads to a lot of grey area.**

Thank you for raising this point. After due reflection, we feel that as we have expanded the scope of the manuscript and the title to include the impact of our nutritional intervention on the food security of TB households during the pandemic lockdowns, we believe this sentence is more relevant now.

**87: Referencing the study population to another study which describes the patient with TB and their household contacts may not be sufficient.**

Due to space constraints, we have cited the TB LION protocol paper, which provides a detailed description of the study population of this current study. We have amended the language to be more descriptive (lines 94-97):

*“In brief, approximately 60% of persons with TB in this region are attributable to undernutrition. All household contacts are between 18 and 60 years old, do not have diabetes, and live with a person with microbiologically-confirmed TB.”*

**97 : Study population will need some clarity on the demographics of the household contacts you have selected, the age groups, number of the people in the household, as it is difficult to understand the results without having base information about the same.**

We agree, and have added information on the age and gender of the IDI participants, as well as the number of people in the participating households (lines 100-102):

*“Among the household contacts who participated in the IDIs, six were male and 12 were female, with an average age of 30.9 (SD=11.7). On average, participating households had 4-5 people.”*

**118-119: Relevance of the policemen statement without context?**

Thank you. The police statement provides a testament to the severity with which the lockdown was enforced by the police and the deterrents to leaving one’s home that resulted in reduced food access. We have added context in the “Background on the COVID lockdowns” section, lines 130-131.

*“The lockdown measures were strictly enforced by the police.”*

**129 - Duration of the period during which the intervention was stalled must be written, as it a significant confounder!**

We agree, and have added additional information in lines 152-153:

*“The intervention was stalled until October 2020 for two participants and December 2020 for one participant.”*

**Looking forward to reading the revised manuscript with the supporting documents. All the best!**

Thank you so much for your thoughtful review!

**Reviewer #2: This study examines other factors that contributed to the increase in TB mortality reported during the COVID-19 pandemic beyond late TB detection and notification of cases that resulted from disrupted health systems. The authors argued that undernutrition due to food insecurity because of the COVID lockdowns in South India could be an overlooked modifiable factor for increased mortality during this period. They expressed their view in a free flowing and lucid manner that builds on existing body of knowledge from previous studies. These include food insecurity resulting from the COVID-19 pandemic, and effects of undernutrition during periods of food insecurity in increasing TB incidence (due to nutritionally acquired immune deficiency) and unfavorable TB treatment outcomes, including mortality from cavitation and lung involvement, increased risk of drug toxicity, and poor engagement with care due to hunger. The authors highlighted key limitations to the study including small number of participants and response bias that could arise from the participants being inhibited in their responses, since they were beneficiaries of a nutritional intervention program supported by the interviewers.**

Thank you for your insightful comments and suggestions!

**The authors could do a better job of aligning the purpose and result of the study to the tittle. Whereas the title refers to the implications of food insecurity on increased TB mortality in South India, the purpose of the study, as described in the abstract section, points to the impact of COVID lockdowns on food security.**

Thank you for your thoughtful critique. In response, we have updated the title to focus on the impact of COVID-19 on food insecurity in households with TB:

*“Food insecurity in South Indian households with TB during COVID-19 lockdowns and the impact of nutritional interventions: a qualitative study”*

We have however retained the connection between our observations and the larger epidemiological trends during the COVID-19 pandemic in our discussion section.

**In a similar vein, the results section highlights the reduced income, attendant food insecurity and coping strategies, as well as the benefits of nutritional interventions to the participants. There was no supporting data from the study region or participants that explicitly indicated lesser TB mortality rates among beneficiaries of the nutritional interventions. At best, the inference between food insecurity and increased mortality, is based on other studies that indicated reduced TB incidence from improved macro and micro nutrition (the RATIONS trial) and unfavorable treatment outcome from undernutrition (the LION study). The authors should consider adding supportive data to indicate lower TB mortality among the study population that benefitted from the nutritional supplementation.**

Thank you for your comment. To be clear, our goal in this study is to provide an in-depth look at the household-level food insecurity in households of PWTB, their coping mechanisms, and the impact of our nutritional intervention. Unlike the RATIONS study, the TB LION study is focused on understanding the immunological impact of nutritional supplementation and is also not powered to assess the impact of nutritional support on TB incidence and mortality. A recent cluster-randomized study from Odisha, India by Mahapatra et al. (2024) has demonstrated the beneficial impact of nutritional support on improving TB treatment outcomes.

We have revised the draft to reflect this in lines 232-243.

*“Fortunately, these risks can be mitigated in large part through food security interventions. While the TB LION is focused on exploring the immunological impacts of nutritional support, larger studies in India have established a causal link between improved food access and reductions in TB disease incidence by providing macronutrient and micronutrient support through food baskets [18]. The Indeed, the RATIONS trial demonstrated that providing food baskets reduced TB disease incidence by approximately 40% among household contacts of persons with TB disease. Persons with TB in the RATIONS trial also had higher weight gain and possibly lower mortality compared to previous cohorts [7, 19]. More recently, a cluster-randomized study by Mahapatra et al. in Odisha, India demonstrated that nutritional support improved treatment success by approximately 40% [20]. The INSTITUT study in Benin and Togo is currently studying the impact of nutritional support in a West African population [21].*
